# Neonatal Sucrose and Internalizing Behaviors at 18 Months in Children Born Very Preterm

**DOI:** 10.1001/jamanetworkopen.2025.4477

**Published:** 2025-04-10

**Authors:** Mia A. McLean, Manon Ranger, Jeffrey N. Bone, Thiviya Selvanathan, Stephanie H. Au-Young, Cecil M. Y. Chau, Vann Chau, Linh Ly, Edmond Kelly, Anne Synnes, Steven P. Miller, Ruth E. Grunau

**Affiliations:** 1 Department of Psychology and Neuroscience, Auckland University of Technology, Auckland, New Zealand; 2 Department of Pediatrics, University of British Columbia, Vancouver, British Columbia, Canada; 3 BC Children’s Hospital Research Institute, Vancouver, British Columbia, Canada; 4 School of Nursing, The University of British Columbia, Vancouver, British Columbia, Canada; 5 Department of Pediatrics, Division of Neurology, The Hospital for Sick Children and University of Toronto, Toronto, Ontario, Canada; 6 Department of Pediatrics, Division of Neonatology, The Hospital for Sick Children and University of Toronto, Toronto, Ontario, Canada; 7 Department of Pediatrics, Division of Neonatology, Mount Sinai Hospital, Toronto, Ontario, Canada

## Abstract

**Importance:**

In the neonatal intensive care unit (NICU), neonates born very preterm (<33 weeks’ gestation) are exposed to great numbers of painful procedures, which is associated with more internalizing (anxiety and depressive) behaviors later in childhood. Oral sucrose is commonly used in NICUs to treat acute procedural pain and is effective in reducing behavioral responses, but the long-term associations between oral sucrose and child behaviors have not yet been examined.

**Objective:**

To examine associations between cumulative neonatal pain and sucrose exposure in early life (prior to approximately 32 weeks postmenstrual age), in relation to child behaviors (internalizing and externalizing) at 18 months corrected age (CA) in children born very preterm and to examine whether the associations are sex specific.

**Design, Setting, and Participants:**

In a prospective, longitudinal cohort study, children born very preterm (24-32 weeks’ gestational age [GA]) were recruited from 2015 to 2019 from 3 tertiary NICUs in Canada and attended a follow-up visit at 18 months CA. Data analysis was performed from February to May 2024.

**Exposures:**

The clinical protocol to treat acute procedural pain at site 1 was facilitated tucking with nonnutritive sucking; sites 2 and 3 used 24% sucrose with nonnutritive sucking. Prospective clinical record review was conducted (eg, number of painful procedures, cumulative sucrose dose, analgesia, sedation, days receiving mechanical ventilation, and surgical procedures).

**Main Outcomes and Measures:**

Parents reported on their child’s behavior on the Child Behavior Checklist (CBCL; 1.5 to 5 years) yielding internalizing and externalizing scores.

**Results:**

In total, 192 children (110 male [57%]) were included in the current study. After applying propensity score weights to adjust for clinical factors across sucrose and nonsucrose sites and accounting for neonatal pain, cumulative sucrose (milliliters) in early life was not associated with internalizing scores (*B* = 0.62; 95% CI, −0.46 to 1.99). However, greater neonatal pain exposure was significantly associated with higher 18-month CBCL internalizing scores (*B* = 0.01; 95% CI, 0.0003 to 0.0135; *R*^2^ = 1.8%). There were no associations with externalizing scores, and associations were not moderated by child sex.

**Conclusions and Relevance:**

In this cohort study of children born very preterm across 3 tertiary NICUs in Canada, cumulative sucrose exposure in early life demonstrated no association with child behavior and did not ameliorate the association between greater neonatal pain and internalizing behaviors. Further research is needed to identify pain management strategies that can effectively mitigate or protect against adverse behavioral outcomes in children born very preterm.

## Key Points

**Question:**

Does sucrose treatment for minor neonatal acute procedural pain ameliorate the poorer child behaviors associated with neonatal pain in children born very preterm?

**Findings:**

In this cohort study of 192 children born very preterm (<33 weeks’ gestation), higher neonatal pain exposure, but not cumulative sucrose receipt in early life, was associated with greater internalizing behaviors at age 18 months. The findings were specific to internalizing behaviors, and associations did not differ by child sex.

**Meaning:**

These findings suggest that sucrose for the management of neonatal pain does not ameliorate the association between greater neonatal pain and internalizing behaviors in children born very preterm, and further research on pain management strategies is needed.

## Teaser

This cohort study examines associations of cumulative neonatal pain and sucrose exposure in early life with behavioral problems at age 18 months among children born very preterm.

## Introduction

Children born very preterm (24-32 weeks’ gestational age [GA]) undergo frequent painful invasive procedures (approximately 10 per day)^1,2^ while hospitalized in the neonatal intensive care unit (NICU).^3^ Cumulative neonatal pain^2,4,5,6^ (number of invasive procedures) has been adversely associated with altered brain structure in early life^7,8,9^ and at school age^10,11^ and with neurodevelopment across childhood,^12^ including internalizing (anxiety and depressive) behaviors.^13,14,15^ Internalizing behaviors are highly prevalent,^16,17^ and parents consider behavior a top priority for health improvement.^18^

Oral sucrose is the most commonly used treatment in NICUs to manage acute procedural pain because it yields observable calming effects on infant pain behaviors.^19,20^ However, administration of sucrose primarily decreases behavioral rather than physiological and cortical pain responses.^20^ Although the mechanisms of action remain unclear, animal studies support the activation by intraoral sucrose of key brainstem sites involved in pain modulation.^21^ Recent work in a neonatal mouse model showed long-term adverse outcomes on brain structure^22^ and memory^23^ from early repetitive exposure. Given these preclinical research findings, the widespread use of oral sucrose and the heightened susceptibility to external stressors on the immature very preterm brain,^24^ there is concern regarding possible long-term adverse outcomes of repetitive use of sucrose in human neonates.^25,26,27^ Close to a decade since the American Academy of Pediatrics Committee on Fetus and Newborn and Section on Anesthesiology and Pain Medicine in 2016^28^ raised concerns, there remains a dearth of research investigating associations between repetitive sucrose administration and long-term outcomes.^17^ The limited evidence suggests that sucrose may not provide additional benefit above maternal-newborn skin-to-skin contact (SSC) for neurobehavioral outcomes at term-equivalent age,^29^ but that high sucrose exposure during the first week of life may be associated with poorer neurobehavior at term-equivalent age, for children born at less than 31 weeks’ gestation.^30^ Following prior work from our group showing painful exposures in early-life (up to approximately 32 weeks postmenstrual age [PMA]) are most associated with altered brain development,^7,8,9^ we found that early-life sucrose exposure exacerbated the association between greater neonatal pain and poorer cognition and language at 18 months corrected age (CA).^31^ Moreover, also in very preterm infants, Schneider et al^32^ found both greater pain and glucose exposure (another sweet-tasting solution) were associated with slower neonatal brain growth, particularly in female children, as well as poorer neurodevelopment at age 18 months CA.

In a previous study^14^ using data from the current longitudinal prospective cohort of children born very preterm recruited from 3 tertiary NICUs in Canada, we examined neonatal cumulative pain and sucrose exposure in early life (approximately 32 weeks PMA) in association with child behaviors (internalizing and externalizing) at 18 months CA. Children born very preterm compared with those born full-term displayed elevated internalizing but not externalizing behaviors at 18 months CA (mean difference, approximately 4 T-scores^14,33^) and in childhood (effect size of 0.2 across studies).^14^ A recent meta-analysis^17^ found a greater than 2-fold risk for anxiety among children born preterm compared with their full-term peers across ages 3 to 19 years. Importantly, for internalizing behaviors assessed by validated parent report measures such as the Child Behavior Checklist (CBCL),^34^ onset in toddlerhood is associated with later problems.^13,35,36,37^ Therefore, we hypothesize that associations of these exposure in the NICU will be specific to internalizing behaviors. Furthermore, we examined whether associations differed by sex, given prior work demonstrating female-specific effects of sweet-tasting solutions in animal models^38^ and clinical research,^32^ supported by evidence of female-specific associations among neonatal pain and brain structure,^32^ function,^39^ and behavior.^40^

## Methods

### Study Population

This study is part of a larger multicenter prospective longitudinal cohort study^14^ across 3 tertiary NICUs in Canada. Neonates born very preterm (24-32 weeks plus 6 days GA) were recruited from the level III NICUs at the Hospital for Sick Children and Mount Sinai Hospital in Toronto, and the BC Women’s Hospital in Vancouver, Canada. Research ethics boards at each site approved the study. Neonates with clinical evidence of a major congenital malformation or syndrome and/or antenatal infection were not eligible for the study. Recruitment took place from April 2015 to April 2019 at Hospital for Sick Children and Mount Sinai Hospital, and from February 2016 to October 2019 at BC Women’s Hospital. We made every effort to approach all families who met the inclusion criteria. In the current study, we include children with parent-completed Child Behavior Check List (CBCL 1.5-5 years) at 18 months CA follow-up visit. Parental written informed consent was obtained at study enrollment and again at 18 months CA. This study followed the Strengthening the Reporting of Observational Studies in Epidemiology (STROBE) reporting guidelines for cohort studies.^41^

### Measures

#### Participant Clinical Characteristics

Trained neonatal research nurses conducted detailed prospective day-by-day medical record review. In this study, we included clinical data across early life as in our prior work,^31^ defined as birth to first imaging on magnetic resonance imaging (at a median [IQR] of 33.0 [29.0-34.4] weeks’ PMA) or to 32 weeks plus 6 days PMA for neonates without this magnetic resonance imaging.

Neonatal pain was operationalized as the number of invasive and noninvasive procedures across early life. Each attempt at a procedure was considered (eTable 1 in Supplement 1).

Cumulative sucrose was calculated as total sucrose in milliliters administered during early life for sites 2 and 3, where sucrose is routinely administered with or without nonnutritive sucking (ie, used if the neonate was able to hold the pacifier securely to induce nonnutritive sucking; 0.1 mL/dose of 24% solution). Sucrose dose is measured by premeasured syringes prepared by the internal pharmacy or nurses draw them up with syringes, with doses recorded by a research nurse. Following the site 1 clinical pain management protocol, sucrose was not administered to very preterm neonates; instead, site 1 used facilitated tucking with nonnutritive sucking for procedures (eAppendix 1 in Supplement 1 for institutional neonatal pain protocols). We created a categorical index of sucrose exposure in early life, coded as none or any.

Cumulative morphine, fentanyl, and midazolam were calculated (intravenous dose plus converted oral dose when applicable) as the average daily dose adjusted for daily body weight, multiplied by the number of days the medication was given during the early-life window. The number of days the neonate received any type of respiratory support (eg, mechanical ventilation, continuous positive airway pressure, or bilevel positive airway pressure) across early life was also calculated.

Numbers of infections were obtained through the Canadian Neonatal Network database. Postnatal culture-positive infection was defined as any positive blood or cerebrospinal culture, necrotizing enterocolitis stage 2 or higher, or retinopathy of prematurity requiring treatment with laser or anti–vascular endothelial growth factor therapy. Major surgical procedures were defined as those requiring laparotomy, thoracotomy, ostomy, or extracorporeal membrane oxygenation or procedures involving the central nervous system across early life. Because few neonates had more than 1 surgery or infection in early life, these variables were coded as none vs any.

#### Child Behavior

The CBCL (1.5 to 5 years)^34^ questionnaire was completed by the primary caregiver at the 18-month CA follow-up visit. We used the CBCL Internalizing (Emotionally Reactive, Anxious/Depressed, Somatic Complaints, or Withdrawn) and Externalizing (Attention Problems or Aggressive Behavior) T-scores. Higher scores represent greater problems (mean [SD] score, 50 [10]; range, 0-100); a T-score of 64 or higher is the clinical problem cutoff for each subscale.

### Statistical Analysis

Data analysis was performed from February to May 2024, conducted in R statistical software version 4.2.1 (R Project for Statistical Computing).^42^ Outliers (>3 SD from the mean) on neonatal pain and sucrose were winsorized.

To adjust for confounding clinical factors across sites, we used inverse probability of treatment weighting propensity score analysis^43^ via R package WeightIt^44^ to specifically balance our sample by sucrose site, comparing sites that administered sucrose for acute pain with site 1, which did not (eTable 2 in Supplement 1). Some infants in NICU sites that administer sucrose as their pain management strategy did not receive sucrose; therefore, we also ran analyses weighted by sucrose exposure (none vs any; eTable 3 in Supplement 1). Further details are provided in eAppendix 2 in Supplement 1.

We ran separate linear regression models accounting for sampling weights to examine whether sucrose and neonatal pain were associated with child internalizing and externalizing T-scores on the CBCL at 18 months CA. We tested the interaction between sucrose and pain in association with child behavior, again using inverse probability of treatment weighting to account for clinical factors. We also tested whether associations differed by child sex (neonatal pain by sex, sucrose by sex, and neonatal pain by sucrose by sex). Model 95% CIs were calculated via the nonparametric bootstrap with 10 000 resamples, and significance was determined using 2-sided 95% CIs, with significance defined as 95% CIs that did not include the null value of zero.

## Results

### Cohort Description

A total of 297 very preterm neonates were enrolled across the 3 sites, and of the 277 eligible for this study, 192 children (110 male [57%]) had parent-reported CBCL 1.5 to 5 years at 18 months CA (eFigure in Supplement 1). Participants missing data at 18 months CA did not differ from those with complete data on neonatal or sociodemographic variables (eTable 4 in Supplement 1).

The Table displays clinical factors and outcome variables by NICU site. Neonates at NICU site 3 were exposed to more neonatal pain than those at site 1 and site 2. Of the 2 sites that administer sucrose, cumulative sucrose was greater at NICU site 3 than site 2; 90 of 114 neonates (79%) received sucrose, and there was a moderate correlation between neonatal pain and cumulative sucrose (*r* = 0.45; *P* < .001). Child internalizing and externalizing behavior T-scores were similar across sites. Those born at a later GA underwent fewer painful procedures (*r* = −0.72; *P* < .001) and received less cumulative sucrose (*r* = −0.30; *P* < .001). See eTable 5 in Supplement 1 for clinical factors and outcome variable data for male and female children.

**Table.** Neonatal Clinical Factors in Early Life and Child Behavior at 18 Months Corrected Age, by Neonatal Intensive Care Unit Site

Characteristic	Mean (SD) [range]		
	No sucrose: site 1 (n = 78)	Sucrose	
		Site 2 (n = 76)	Site 3 (n = 38)
Child sex, No. (%)			
Female	32 (41)	30 (39)	20 (53)
Male	46 (59)	46 (61)	18 (47)
Birth weight, g	1092.1 (350.0) [520.0-1970.0]	1015.8 (347.4) [500.0-1790.0]	1003.8 (365.2) [510.0-2270.0]
Gestational age at birth, wk	28.1 (2.0) [24.0-32.6]	27.7 (2.5) [24.0-31.6]	27.1 (2.2) [23.3-31.1]
Sucrose exposure, No. (%)	NA	56 (73.7)	34 (89.5)
Cumulative sucrose, mL	NA	1.2 (1.3) [0.0-6.4]	4.3 (3.4) [0.0-9.5]
Median (IQR)	NA	0.9 (0.0-2.0)	3.9 (1.0-6.9)
Neonatal pain, No. of painful procedures	168.8 (175.5) [43.0-788.0]	241.3 (228.4) [36.0-935.0]	334.6 (288.8) [52.0-935.0]
Median (IQR)	99.0 (71.2-169.8)	151.0 (82.2-291.0)	163.5 (114.2-435.8)
Respiratory support, d	47.6 (36.2) [0.0-149.0]	66.05 (47.0) [1.0-172.0]	65.5 (51.8) [2.0-186.0]
Median (IQR)	40.0 (21.2-66.8)	62.0 (27.0-103.8)	52.0 (20.2-101.2)
Cumulative fentanyl, µg^a^	40.9 (215.9) [0.0-1598.8]	24.3 (105.2) [0.0-682.7]	82.0 (162.6) [0.0-606.0]
Median (IQR)	0.0 (0.0-2.0)	0.0 (0.0-1.9)	3.5 (1.3-31.3)
Cumulative morphine, mg^a^	2.9 (8.1) [0.0-39.0]	0.1 (0.4) [0.0-2.5]	1.4 (3.3) [0.0-17.5]
Median (IQR)	0.0 (0.0-0.4)	0.0 (0.0-0.0)	0.1 (0.0-1.2)
Cumulative midazolam, mg^a^	1.3 (4.8) [0.0-33.7]	0.0 (0.0) [0.0-0.1]	0.0 (0.0) [0.0-0.1]
Median (IQR)	0.0 (0.0-0.0)	0.0 (0.0-0.0)	0.0 (0.0-0.0)
≥1 Postnatal infection, No. (%)^b^	16 (21)	19 (25)	12 (32)
≥1 Major surgical procedure, No. (%)	6 (8)	1 (1)	12 (32)
CBCL internalizing T-score	43.8 (10.0) [29.0-65.0]	44.3 (9.2) [29.0-72.0]	46.7 (11.9) [29.0-79.0]
Median (IQR)	43.0 (37.0-50.8)	43.0 (37.0-49.5)	45.0 (38.0-53.0)
CBCL externalizing T-score	44.6 (10.2) [28.0-76.0]	45.2 (8.4) [28.0-67.0]	46.2 (9.9) [28.0-70.0]
Median (IQR)	44.0 (37.0-50.8)	46.0 (39.0-51.0)	45.0 (39.2-54.0)

Abbreviations: CBCL, Child Behavior Checklist; NA, not applicable.

^a^ Refers to cumulative dose adjusted for daily body weight.

^b^ Refers to culture-positive infection.

### Child Internalizing Behaviors

#### Weighting by Study Site

After applying propensity weights to account for relevant clinical factors that differed by study site (eTable 2 in Supplement 1) and neonatal pain, absolute standardized mean differences were 0, suggesting adequate balance of covariates. Sucrose exposure (none vs any) was not associated with internalizing scores (*B* = 2.36; 95% CI, −2.74 to 7.57). Cumulative sucrose (milliliters) was also not associated with internalizing scores (*B* = 0.62; 95% CI, −0.46 to 1.99).

After applying propensity weights to account for relevant clinical factors that differed by study site (eTable 2 in Supplement 1; adjusted absolute standardized mean differences equal to zero), greater neonatal pain was associated with higher internalizing scores (*B* = 0.01; 95% CI, 0.0003 to 0.0135; *R*^2^ = 1.8%). Cumulative sucrose was not associated with internalizing scores (*B* = 0.41; 95% CI, −0.52 to 1.41) accounting for neonatal pain. Neonatal pain and cumulative sucrose were not associated with internalizing scores (*B* = 0.00; 95% CI, −0.00 to 0.00). Associations did not differ by child sex (eAppendix 3 in Supplement 1).

#### Weighting by Exposure to Early Sucrose

The same associations were evident when analyses were weighted by clinical factors that differed by sucrose exposure (eTable 3 in Supplement 1). See eAppendix 3 in Supplement 1 for complete reporting.

Our intention-to-treat (weighted by site) and per-protocol (weighted by exposure) models demonstrate reasonable precision. Given a standard error of measurement (SEM) of 3.32 for internalizing, point estimates indicate no clinically meaningful difference in internalizing behaviors for children exposed to sucrose (*B* = 2.38) or cared for at a site administering sucrose (*B* = 2.36). The SEM is a commonly used statistic for assessing individual change on health-related instruments^42^ and clinically meaningful change following treatment.^43^ On the basis of an SD of 10 and Cronbach α = 0.89,^31^ the SEM for the CBCL internalizing subscale is 3.32 T-scores; the SEM for CBCL externalizing subscale is 2.83 T-scores (SD = 10; Cronbach α = 0.9231). One SEM corresponds to a minimal clinically important intraindividual change.^44,45^ Here, we use the SEM as an indication of a clinically meaningful difference in CBCL T-scores between sucrose and nonsucrose sites.

However, the 95% CIs rule out the benefit of sucrose for internalizing behaviors: lower bounds of the 95% CIs indicate no clinically meaningful benefit, and upper bounds of the 95% CIs indicate a clinically important increase in T-scores for children exposed to sucrose or cared for at a site administering sucrose (1-fold to 2-fold the minimally important change). Moreover, point estimates demonstrate that the effect size of cumulative sucrose exposure was larger than that of pain: for every painful procedure, T-scores increased by 0.01, whereas every dose of sucrose (0.1 mL per procedure) was associated with increased internalizing T-scores by 0.041 (weighted by sucrose site) to 0.043 (weighted by sucrose exposure).

### Child Externalizing Behaviors

Regardless of weighting by study site or sucrose exposure, no significant associations or interactions among neonatal pain, sucrose (sucrose exposure, or cumulative sucrose), and externalizing T-scores were evident, and associations did not differ by child sex. See details in eAppendix 3 in Supplement 1.

## Discussion

In a 3-site Canadian prospective longitudinal cohort study of children born very preterm, we investigated the associations between exposure to repetitive sucrose for acute procedural pain during early life and child internalizing and externalizing behaviors at 18 months CA. As hypothesized, the associations were specific to internalizing behaviors. After accounting for site differences in clinical factors, greater neonatal pain (number of painful procedures) was associated with greater internalizing at 18 months CA. Cumulative sucrose exposure in early life was not associated with child internalizing behaviors. No interactive associations between pain and sucrose exposures were evident, and there were no differences between male and female children. Our findings indicate that cumulative sucrose exposure was not associated with better behavioral outcomes at age 18 months in a cohort of children born very preterm. Moreover, cumulative sucrose exposure did not mitigate or exacerbate the negative association between neonatal pain in early life and internalizing behaviors in very preterm children.

Previous clinical studies showed adverse outcomes of greater exposure to sucrose (or glucose) in early life on short-term^30^ and longer-term^31,32^ neurodevelopment in very preterm children. However, those studies did not examine behaviors. We previously found in this cohort of preterm children, that sucrose may exacerbate negative associations of pain with language and cognition at 18 months CA.^31^ Taken together, our work suggests that sucrose may exacerbate negative outcomes of pain on cognitive outcomes, with no association with internalizing behaviors, at least in toddlerhood. Importantly, point estimates and bootstrapped 95% CIs demonstrated no clinically meaningful improvement in internalizing behaviors, with clinically important increases in internalizing related to sucrose exposure most likely. This conclusion is partly supported by our preclinical work. In a mouse model that closely mimics the early pain exposure in preterm neonates in the NICU, we found that neonatal oral sucrose (irrespective of pain exposure) negatively affected brain volumes of limbic white matter (eg, fimbria and stria terminalis) and gray matter structures (eg, hippocampus).^22^ These structures are known to be implicated in the hypothalamic-pituitary-adrenal axis or stress system and regulation of anxiety and stress.^45^ Although we showed in mice that early pain and/or sucrose had deleterious associations with memory as a cognitive function, when mice reached adulthood, we found no association with anxiety,^23^ consistent with the present clinical study in human children. Our preclinical research findings, in conjunction with the clinical studies, raise serious concerns about repetitive sweet taste solutions in association with early brain development, cognition, and behavior. Longer follow-up of very preterm children is needed beyond the second year of life. Child internalizing behaviors are first evident in toddlerhood and persist through school age^13^; thus, it is essential to examine how sucrose is associated with these behaviors through adolescence and beyond.

Our findings highlight that despite consistent evidence demonstrating the effectiveness of sucrose at lowering behavioral pain indicators,^19^ it does not appear to ameliorate or prevent adverse effects of neonatal pain on later anxiety or depressive behaviors^13,14,15^ in children born very preterm, and may be associated with poorer behavior. It is important to mention that the effect size of a single dose of sucrose on internalizing was 4-fold that of 1 painful procedure. Oral sucrose has observable calming effects on human neonate pain behavioral scores^19^ but may have more sedative than analgesic effects.^46,47,48^ Importantly, physiological stability was not improved, nor was stress response (cortisol level) reduced by treating procedural pain with sucrose in very preterm neonates during the first week of life^46^ and was shown to increase adenosine triphosphate utilization and oxidative stress.^48^ Furthermore, nociceptive-regional electroencephalogram responses were not dampened by sucrose administration in preterm neonates undergoing a heel lance, despite pain behaviors being reduced.^47^ Taken together, our findings highlight the need for rigorous short-term and long-term clinical trials to determine the best nonpharmacologic pain management strategy to protect the developing brain during acute routine painful procedures and determine the long-term outcomes of repetitive use of sucrose. Another important issue is that sucrose administered during a period of rapid development of all systems may have outcomes beyond the brain. A recent preclinical study^38^ in a nonpain context in neonatal mice found lower growth in adult female mice (but not male mice) that were repetitively exposed to small amounts of sucrose during the first week of life (same sucrose protocol to preclinical studies examining long-term effects of pain and sucrose^22,23^), as well as lower serum insulin-like growth factor–1, compared with fructose, glucose, or water. In addition, altered liver metabolism was found in both female and male mice exposed to neonatal sucrose. These findings highlight the importance of examining biomarkers, not just neurodevelopmental and behavioral outcomes.

Higher neonatal pain in early life was associated with greater internalizing behaviors in children born very preterm at 18 months CA. In a series of studies across 3 cohorts of Canadian children born very preterm, we have demonstrated that exposure to neonatal pain–related stress is associated with widespread difficulties in neurobehavioral outcomes, including cognition, motor skills, and internalizing behavior in infancy through school age.^12,13,49^ Despite dramatic changes in the last 15 to 20 years in clinical care and the growing awareness of the adverse neurodevelopmental impact of pain in preterm newborns, the long-term negative effects of neonatal pain were again evident in this latest multicenter cohort, born between 2015 and 2019. Studies in Europe have found similar associations between neonatal pain and brain development,^50,51^ hypothalamic-pituitary-adrenal functioning,^6^ and neurodevelopment^52^ in very preterm cohorts.

### Limitations and Strengths

In early 2024, The Canadian Paediatric Society issued a position statement on SSC for full-term and preterm newborns emphasizing its beneficial effects on pain regulation and neurodevelopment.^53^ This statement highlighted, among other important beneficial effects, the efficacy of SSC for reducing procedural pain in preterm newborns and infants,^54^ especially with breastfeeding as a stress-regulating strategy.^55^ Other recommendations included documenting SSC in the child’s hospital medical record. Moreover, there has been substantial uptake of family-centered care approaches in NICUs across Canada following a large-scale randomized clinical trial^56^ showing beneficial long-term outcomes on child behavior.^57^ Our current study protocol did not include data about each site’s developmental care and other neuroprotective care procedures (commonly referred to as *bundles*). A limitation of our study is that we were not able to collect data on the administration of other nonpharmacological interventions such as SSC, which is encouraged across study sites, despite our efforts to record parental presence, since this was not routinely documented. Furthermore, we were unable to accurately collect data on the use of facilitated tucking at site 1, which is their first-line nonpharmacological pain management strategy for procedures. Future studies examining the long-term outcomes of neonatal pain and sucrose exposure should include SSC time, family presence or involvement, and all other procedural pain management strategies used within the study sites so that all clinical care practices are accurately represented and, thus, taken into account in statistical analyses. It is notable that the average cumulative sucrose dose across NICU stay in sites 2 and 3 was proportional to that reported in tertiary NICUs across Canada for length of NICU stay (average of 5.5 mL per neonate during NICU admission).^58^ At the sites administering sucrose, only 79% of neonates received sucrose, and the correlation between cumulative sucrose exposure and neonatal pain was moderate, resulting in greater certainty of findings in per-protocol vs intention-to-treat analyses. Our current findings and those of a recent Cochrane review^19^ suggest that the need for trials investigating efficacious neonatal pain management strategies is urgent, for short-term and long-term well-being of children born very preterm. Additional limitations include lack of accounting for the family history of anxiety or depression and race or ethnicity. Despite the potential for parent-reported bias of child behavior, the CBCL is widely regarded as a reference standard measure for assessing emotional and behavioral problems in children and is the standardized measure of child behavior used across the Canadian Neonatal Follow-up Network.

A major strength of our study is that it was conducted across 3 major tertiary NICUs, each using different pain-management protocols. This diversity allowed us to include a comparison that had no exposure to sucrose. Another strength of our study is that we applied a robust statistical technique, propensity score weighting, to account for differences in primary clinical factors, including potential effects of pharmacological pain medications (eg, morphine, fentanyl, or midazolam), across study sites. Given mixed findings as to the potential associations of pharmacological medications with long-term outcomes,^26^ future research should explore these associations. Such studies, using larger samples, could compare long-term outcomes of sucrose for those born earliest (extremely low gestational age [24-28 weeks]) with those born at very low gestational age (29-32 weeks’ gestation), to better inform susceptibility models and intervention delivery.

## Conclusions

In a multisite Canadian prospective longitudinal cohort of children born very preterm, sucrose did not mitigate the well-established negative association between neonatal pain and child internalizing behaviors. Moreover, we highlight that, at best, sucrose is not associated with any benefits for behavior in this population and, at worst, may be associated with clinically meaningful increases in internalizing behaviors. Our findings are concerning and highlight the need for further work to find the safest and most effective nonpharmacological treatments for very preterm newborns. Providing brain-protective, evidence-based treatment for procedural pain in very preterm newborns remains a top priority in neonatal care.

## Notes

Supplement 1. **eTable 1.** Invasive and non-invasive procedures **eAppendix 1.** Institutional neonatal pain protocols **eTable 2.** Comparison of clinical and demographic factors across early-life as a function of sucrose and no sucrose sites **eTable 3.** For all sites comparison of clinical factors across infants exposed to sucrose versus those who did not receive any in early-life **eAppendix 2.** Statistical analysis **eFigure.** Participant flow chart **eTable 4.** Comparison of observed characteristics between participants with complete and incomplete data **eTable 5.** Neonatal clinical factors in early-life and 18-month CA child behavior as a function of child sex **eAppendix 3.** Supplemental results

Supplement 2.
Data Sharing Statement
