# Supplementary material for: Neonatal Sucrose and Internalizing Behaviors at 18 Months in Children Born Very Preterm
Source: JAMA Netw Open. 2025 Apr 10;8(4):e254477. doi: 10.1001/jamanetworkopen.2025.4477 (PMC11986768; doi:10.1001/jamanetworkopen.2025.4477)
Supplement: Supplement 1. — eTable 1. Invasive and non-invasive procedures eAppendix 1. Institutional neonatal pain protocols eTable 2. Comparison of clinical and demographic factors across early-life as a function of sucrose and no sucrose sites eTable 3. For all sites comparison of clinical factors across infants exposed to sucrose versus those who did not receive any in early-life eAppendix 2. Statistical analysis eFigure. Participant flow chart eTable 4. Comparison of observed characteristics between participants with complete and incomplete data eTable 5. Neonatal clinical factors in early-life and 18-month CA child behavior as a function of child sex eAppendix 3. Supplemental results [file jamanetwopen-e254477-s001.pdf]

Supplemental Online Content

McLean MA, Ranger M, Bone JN, et al. Neonatal sucrose exposure and internalizing behaviors at 18 months in children born very preterm. *JAMA Netw Open*. 2025;8(4):e254477.
doi:10.1001/jamanetworkopen.2025.4477

eTable 1. Invasive and non-invasive procedures

eAppendix 1. Institutional neonatal pain protocols

eTable 2. Comparison of clinical and demographic factors across early-life as a function of sucrose and no sucrose sites

eTable 3. For all sites comparison of clinical factors across infants exposed to sucrose versus those who did not receive any in early-life

eAppendix 2. Statistical analysis

eFigure. Participant flow chart

eTable 4. Comparison of observed characteristics between participants with complete and incomplete data

eTable 5. Neonatal clinical factors in early-life and 18-month CA child behavior as a function of child sex

eAppendix 3. Supplemental results

This supplemental material has been provided by the authors to give readers additional information about their work.

© 2025 McLean MA et al. *JAMA Network Open*.

eTable 1. Invasive and non-invasive procedures

Invasive	Non-Invasive
Peripheral arterial line stab	PICC dressing change
Peripheral IV insertion	PICC removal
PICC insertion	IV burns
Venous blood draw	IV burns treatment
Heel poke	Penrose drain removal
IM injection + SC injection	Ostomy change
Chest tube insertion	ETT retape
Pleural tap	ETT suction
Penrose drain insertion	NP/OP suction
Paracentesis	Umbilical artery/Umbilical vein catheter insertion
Intubation	Urinary catheter insertion
Suprapubic tap	OG insertion
LP reservoir	OG removal
Circumcision	NG/NJ/ND insertion
	NG/NJ/ND removal
	Dressing change
	EPO
	X-ray
	Eye exam
	Invasive swab
	Pressure sore
	Chest compressions
	US

© 2025 McLean MA et al. *JAMA Network Open*.

eAppendix 1. Institutional neonatal pain protocols

Site 1: The use of oral sucrose for treating mild procedural pain in infants, including injections, IV insertion, venipuncture, heel lance, and clinical procedures, is reserved for when other preferred non-pharmacological methods are not available. Non-pharmacological methods include breastfeeding, skin-to-skin contact, non-nutritive sucking with a facilitated tuck or saddle. Repeated use of oral sucrose is also not advised at this institution. Opioids may be used to treat moderate-severe pain and in general, fentanyl is used for post-operative pain, however this is not part of an institutional guideline and may vary depending on the attending provider.

Site 2: At this institution, non-pharmacologic measures (e.g. breastfeeding) in combination with 0.1mL/dose of 24% oral sucrose may be used to treat mild procedural pain in infants such as for heel lance, intramuscular/subcutaneous injection, venipuncture, arterial puncture, and insertion of peripheral IV. Morphine may be used for some procedures, such as chest drain insertion, insertion of central venous or peripheral arterial catheters in a ventilated patient. Opioids may also be used to treat painful conditions (e.g. NEC) when Premature Infant Pain Profile scores indicate severe pain.

Site 3: 0.1 mL/dose of 24% oral sucrose is routinely used for procedural pain in neonates and young infants at this institution including for IV insertion, venipuncture, heel lance, nasogastric tube insertion, dressing changes, and urinary catheter insertion. The dose may be given prior to a procedure, or at 2-minute intervals throughout the procedure for prolonged procedures. Opioids are used to treat moderate to severe pain and may be required for some procedures, in which case sucrose may be given as an adjunct and not in place of opioids or other analgesic medications.

© 2025 McLean MA et al. *JAMA Network Open*.

eTable 2. Comparison of clinical and demographic factors across early-life as a function of sucrose and no sucrose sites

	Mean (SD)		
	**No Sucrose site** n = 78	**Sucrose sites** n = 114	**SMD** [a]
**Child sex, male n(%)**	46 (59)	64 (56)	.057
**Gestational age at birth (wks)**	28.07 (2.02)	27.47 (2.25)	.278
**Respiratory support (days)**	47.59 (36.19)	65.88 (48.40)	.428
**1+ major surgery, n (%)**	6 (8)	12 (11)	.428
**1 + culture positive confirmed postnatal infection, n (%)**	16 (21)	31 (27)	.157
**Cumulative morphine (mg/kg)** [b]	2.88 (8.09)	0.54 (2.23)	.394
**Morphine, received, n (%)**	28 (35.4)	27 (23.7)	.260
**Cumulative midazolam (mg/kg)** [b]	1.29 (4.79)	0.00 (0.02)	.378
**Midazolam, received, n (%)**	11 (13.9)	4 (3.5)	.376
**Cumulative fentanyl (mcg/kg)** [b]	40.92 (215.92)	43.50 (129.44)	.015
**Fentanyl, received, n (%)**	31 (39.2)	73 (64.0)	.512

[a] Standardised mean difference [b] cumulative dose adjusted for daily body weight

© 2025 McLean MA et al. *JAMA Network Open*.

eTable 3. For all sites comparison of clinical factors across infants exposed to sucrose versus those who did not receive any in early-life

	Mean (SD)		
	**No Sucrose** n = 102	**Sucrose** n = 90	**SMD** [a]
**Child Sex, male n (%)**	64 (63)	46 (51)	.237
**Gestational age at birth (wks)**	28.20 (2.02)	27.17 (2.21)	.485
**Respiratory support (days)**	44.06 (36.12)	74.76 (47.91)	.724
**1+ major surgery, n (%)**	7 (7)	12 (13)	.216
**1 + culture positive confirmed postnatal infection, n (%)**	19 (19)	28 (31)	.292
**Cumulative morphine (mg/kg)** [b]	2.24 (7.16)	0.64 (2.49)	.298
**Morphine, received, n (%)**	33 (32.0)	22 (24.4)	.169
**Cumulative midazolam (mg/kg)** [b]	0.98 (4.22)	0.01 (0.03)	.328
**Midazolam, received, n (%)**	11 (10.7)	4 (4.4)	.237
**Cumulative fentanyl (mcg/kg)** [b]	31.90 (189.27)	54.42 (143.82)	.134
**Fentanyl, received, n (%)**	42 (40.8)	62 (68.9)	.589

[a] standardised mean difference [b] Cumulative dose adjusted for daily body weight

© 2025 McLean MA et al. *JAMA Network Open*.

eAppendix 2. Statistical analysis

Inverse probability of treatment exposure weighting (IPTW) uses weights based on the propensity score to create a pseudo sample in which the distribution of covariates is designed to be independent of treatment assignment (in our study, sucrose administration).[40] Weighting statistical models by propensity scores is sufficient for removing bias associated with observed covariates.[40] Analysis by sucrose-site is analogous to an intention to treat (where sucrose was intended to be given at the given site), while weighting analyses by sucrose exposure (“none” vs “any”) is analogous to a per-protocol effect. We calculated propensity scores using relevant neonatal clinical factors (gestational age at birth, days on respiratory support, morphine, midazolam, fentanyl, presence of infection, major surgery) for both study sites and sucrose exposure analyses, and report standardised mean differences in eTables 2 and 3. Due to the positive skew of cumulative morphine, midazolam and fentanyl, and to ensure best model fit, these variables were recoded “none” versus “any” and labelled morphine exposure, midazolam exposure and fentanyl exposure, respectively.

© 2025 McLean MA et al. *JAMA Network Open*.

eFigure. Participant flow chart

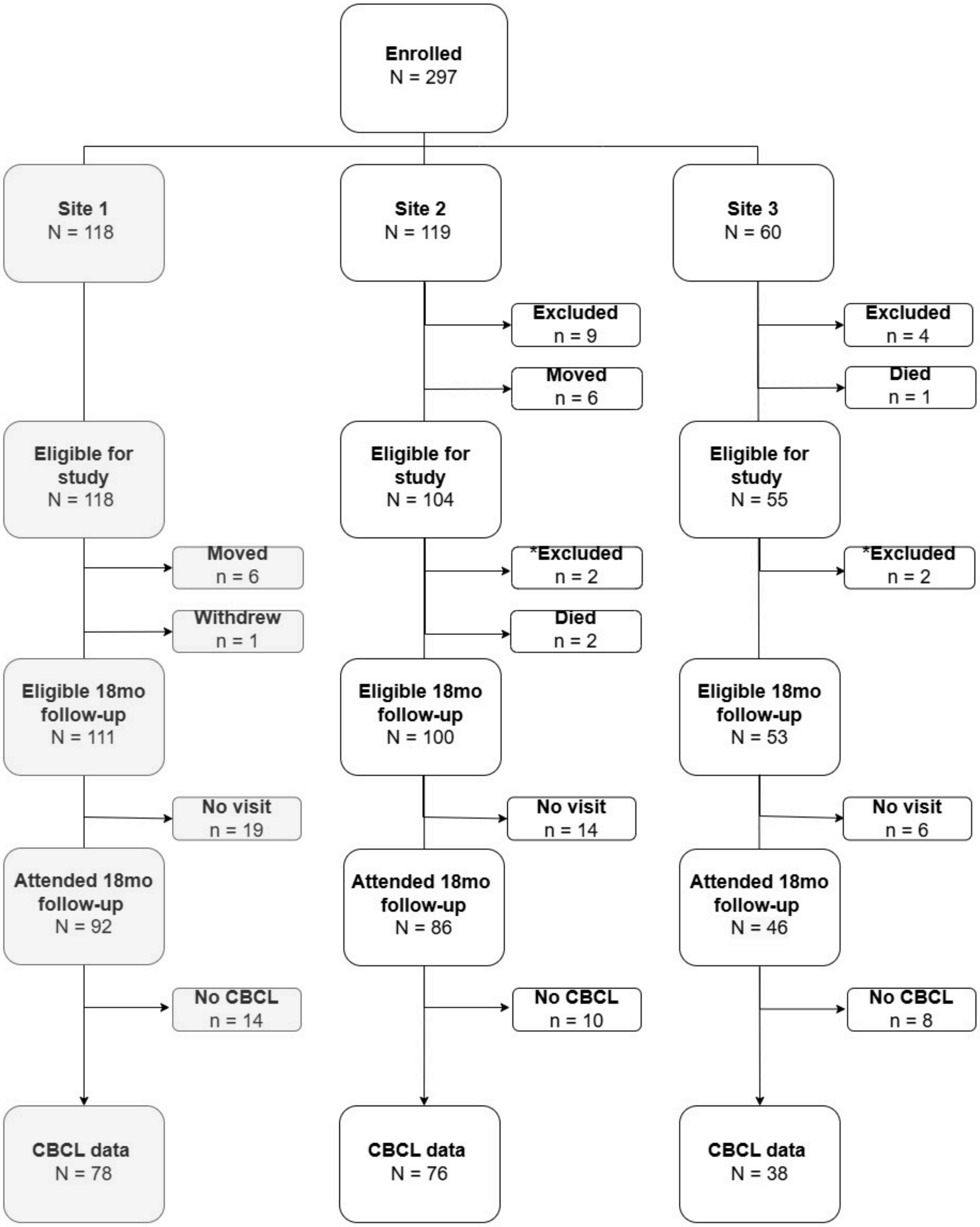

Note: Grey boxes indicate non-sucrose site.
*Site 2 and 3 excluded infants with no neonatal MRI; 1 infant was excluded due to clinical evidence of a congenital malformation or syndrome after scan 1

© 2025 McLean MA et al. *JAMA Network Open*.

eTable 4. Comparison of observed characteristics between participants with complete and incomplete data

	Missing 18-month CBCL data	Complete study data	P value
**Child Sex**			.567
Male	45 (54)	110 (57)	
**Study site**			.646
Site 1	39 (46)	78 (41)	
Site 2	31 (37)	76 (40)	
Site 3	14 (17)	38 (20)	
**Gestational age**			.847
Mean (SD)	27.7 (2.8)	27.7 (2.2)	
Range (min - max)	22.7 - 32.4	24.0 - 32.6	
**Cumulative sucrose, mL**			.082
Mean (SD)	2.4 (6.6)	1.4 (2.7)	
Range (min - max)	0.0 - 50.0	0.0 - 16.5	
**Number of painful procedures**			.175
Mean (SD)	276.6 (286.7)	231.8 (234.5)	
Range (min - max)	24.0 - 1241.0	36.0 - 1042.0	
**Cumulative fentanyl[a] (mcg)**			.887
Mean (SD)	45.7 (189.9)	42.5 (169.4)	
Range (min - max)	0.0 - 1057.2	0.0 - 1598.8	
**Cumulative midazolam [a] (mg)**			.532
Mean (SD)	0.3 (1.9)	0.5 (3.1)	
Range (min - max)	0.0 - 16.3	0.0 - 33.7	
**Cumulative morphine [a] (mg)**			.802
Mean (SD)	1.3 (3.9)	1.5 (5.5)	
Range (min - max)	0.0 - 23.8	0.0 - 39.0	
**Major Surgeries, 1+ n (%)**			.183
	13 (15)	19 (10)	
**Postnatal infection, 1+ [b] n (%)**			.946
1	20 (24)	47 (24)	
**Respiratory support, days**			.770
Mean (SD)	60.2 (51.2)	58.4 (44.7)	
Range (min - max)	0.0 - 200.0	0.0 - 186.0	

© 2025 McLean MA et al. *JAMA Network Open*.

eTable 5. Neonatal clinical factors in early-life and 18-month CA child behavior as a function of child sex.

	M(SD)	
	Female<br>N = 82	Male<br>N = 110
**Study site, n (%)**		
Study site 1	32 (39)	46 (42)
Study site 2	30 (37)	46 (42)
Study site 3	20 (24)	18 (16)
**Birth weight (g)**	990.50 (349.46)	1084.57 (350.76)
**Gestational age (weeks)**	27.44 (2.20)	27.92 (2.14)
**Sucrose exposure, yes**	44 (53.7)	46 (41.8)
**Cumulative sucrose, mL**	1.56 (2.32)	1.18 (2.34)
**Neonatal pain, no. of procedures**	241.87 (233.17)	221.74 (227.94)
**Respiratory support, days**	61.17 (45.93)	56.42 (43.80)
**Major surgeries, 1+, n (%)**	7 (9)	12 (11)
**Postnatal infection, 1+** [b] **n (%)**	22 (27)	25 (23)
**Cumulative fentanyl (mcg)** [a]	45.73 (161.74)	40.01 (175.65)
**Cumulative morphine (mg)** [a]	1.85 (5.85)	1.22 (5.30)
**Cumulative midazolam (mg)** [a]	0.44 (1.69)	0.59 (3.84)
**CBCL Internalizing T-score**	43.96 (9.67)	45.00 (10.43)
**CBCL Externalizing T-score**	44.85 (8.69)	45.34 (10.00)

[a] cumulative dose adjusted for daily body weight, [b] culture positive infection

© 2025 McLean MA et al. *JAMA Network Open*.

eAppendix 3. Supplemental results.

Child Internalizing Behaviors

Weighting by study site. Child sex did not moderate the relationship between neonatal pain and Internalizing scores, *B* = .01, 95%CI [-0.01, 0.02], *p* = .464, or cumulative sucrose exposure and Internalizing scores, *B* = -0.29, 95%CI [-2.24, 1.42], *p* = .808, (female effect = .61, male effect = 0.32). The three-way interaction of Child sex X neonatal pain X cumulative sucrose in relation to Internalizing scores was not significant, *B* = .000, 95%CI [-0.01, 0.007], *p* = .652.

Weighting by sucrose exposure.

After applying propensity weights to account for relevant clinical factors *and neonatal pain* by sucrose exposure (adjusted absolute SMDs equal to zero for all covariates), sucrose exposure ("none" versus "any") was not related to Internalizing scores, *B* = 2.38, 95%CI [-0.87, 5.77]. Cumulative sucrose exposure (mL) was also not related to Internalizing scores, *B* = 0.62, 95%CI [-0.21, 1.60].

After applying propensity weights to account for relevant clinical factors that differed by sucrose exposure (eTable 2), greater neonatal pain was related to higher Internalizing scores, B = 0.01, 95%CI [0.0001, 0.0139], R2 = 2.7%. Sucrose exposure ("none" versus "any") was not related to Internalizing scores (B = 1.65, 95%CI [-1.55, 5.30]), accounting for neonatal pain. Similarly, cumulative sucrose, was not related to Internalizing scores (*B* = 0.43, 95%CI [-0.54, 1.49], accounting for neonatal pain. Neonatal pain and cumulative sucrose did not interact to predict Internalizing scores, *B* = 0.00, 95%CI [-0.002, 0.004]. Importantly, neonatal pain accounted for larger proportion of variance in Internalizing scores at age 18 months CA than that of the intention to treat analysis.

Child sex did not moderate the relationship between neonatal pain and Internalizing scores, *B* = .006, 95%CI [-0.01, 0.03], *p* = .420, or cumulative sucrose exposure and Internalizing scores,

© 2025 McLean MA et al. *JAMA Network Open*.

B = -0.28, 95%CI [-2.65, 2.09], *p* = .804, (female effect = 0.58, male effect = 0.31); the latter interaction accounted for neonatal pain. The three-way interaction of Child sex X neonatal pain X cumulative sucrose in relation to Internalizing scores was not significant, *B* = 0.00, 95%CI [-0.01, 0.01], *p* = .668.

Child Externalizing Behaviors

Weighting by study site. After applying propensity weights to account for relevant clinical factors *and neonatal pain* by sucrose exposure, adjusted absolute SMDs were equal to zero for all covariates. Sucrose exposure (“none” versus “any”) was not related to Externalizing scores, *B* = 1.82, 95%CI [-3.02, 6.59]. Cumulative sucrose exposure was also not related to Externalizing scores, *B* = 0.28, 95%CI [-0.58, 1.13].

After applying propensity weights to account for relevant clinical factors that differed by study site (eTable 2), neonatal pain was not related to Externalizing scores, *B* = .00, 95%CI [-0.00, 0.01], R^2 = 0.4%. Sucrose exposure (“none” versus “any”) was not related to Externalizing scores (*B* = 1.38, 95%CI [-1.93, 4.73]) accounting for neonatal pain. Similarly, when weighted by study site, cumulative sucrose was not related to Externalizing scores (*B* = 0.18, 95%CI [-0.50, 0.87]) accounting for neonatal pain. Neonatal pain and cumulative sucrose did not interact to predict Externalizing scores, *B* = 0.00, 95%CI [-0.00, 0.00].

Child sex did not moderate the relationship between neonatal pain and Externalizing scores, *B* = .00, 95%CI [-0.01, 0.01], *p* = .842, or cumulative sucrose exposure and Externalizing scores, *B* = -0.33, 95%CI [-2.13, 1.37], *p* = .656, *p* = .662 (female effect = 0.31, male effect = 0.03). The three-way interaction of Child sex X neonatal pain X cumulative sucrose in relation to Externalizing scores was not significant, *B* = .00, 95%CI [-0.01, 0.01].

© 2025 McLean MA et al. *JAMA Network Open*.

Weighting by sucrose exposure. After applying propensity weights to account for relevant clinical factors *and neonatal pain* by sucrose exposure (adjusted absolute SMDs equal to zero for all covariates), sucrose exposure (“none” versus “any”) was not related to Externalizing scores, *B* = 1.77, 95%CI [-1.45, 4.87]. Cumulative sucrose exposure was also not related to Externalizing scores, *B* = -0.37, 95%CI [-0.37, 0.88].

After applying propensity weights to account for relevant clinical factors that differed by sucrose exposure (eTable 2), greater neonatal pain was note related to Externalizing scores, *B* = .00, 95%CI [-0.00, 0.01]. Sucrose exposure (“none” versus “any”) was not related to Externalizing scores (*B* = 1.33, 95%CI [-2.12, 4.70]). Similarly, cumulative sucrose was not related to Externalizing scores (*B* = 0.175, 95%CI [-0.49, 0.89]) with neonatal pain in the model. Neonatal pain and cumulative sucrose did not interact to predict Externalizing scores, *B* = 0.00, 95%CI [-0.00, 0.00].

Child sex did not moderate the relationship between neonatal pain and Externalizing scores, *B* = 0.00, 95%CI [-0.01, 0.01], *p* = .846, or cumulative sucrose exposure and Externalizing scores, *B* = -0.26, 95%CI [-2.12, 1.44], *p* = .662 (female effect = 0.35, male effect = 0.06). The three-way interaction of Child sex X neonatal pain X cumulative sucrose in relation to Externalizing scores was not significant, *B* = 0.00, 95%CI [-0.01, 0.01], *p* = .162.

Point-estimates of relationships between pain, sucrose and Externalizing demonstrate differences in T-scores do not meet the minimal clinically important difference of 2.83 T-scores. Lower bound of confidence intervals demonstrate a potential benefit of sucrose exposure on Externalizing scores at age 18 months CA, however intervals are skewed towards an adverse effect, with greater sucrose related to greater Externalizing. Results rule out a large benefit of sucrose, particularly when considering per-protocol analysis (weighted by sucrose exposure).

© 2025 McLean MA et al. *JAMA Network Open*.
