# Supplementary material for: Neonatal Sucrose and Internalizing Behaviors at 18 Months in Children Born Very Preterm
Source: JAMA Netw Open. 2025 Apr 10;8(4):e254477. doi: 10.1001/jamanetworkopen.2025.4477 (PMC11986768; doi:10.1001/jamanetworkopen.2025.4477)
Supplement: Supplement 2. — Data Sharing Statement [file jamanetwopen-e254477-s002.pdf]

Data Sharing Statement

McLean. Neonatal Sucrose and Internalizing Behaviors at 18 Months in Children Born Very Preterm. *JAMA Netw Open*. Published April 10, 2025.
doi:10.1001/jamanetworkopen.2025.4477

Data
Data available: No
